# Plasma-Derived miRNAs as Fluid Biomarkers to Differentiate Alzheimer’s and Frontotemporal Dementia

**DOI:** 10.3390/cimb48060633

**Published:** 2026-06-17

**Authors:** Rosalinda Di Gerlando, Francesca Dragoni, Evelyne Minucchi, Maria Garofalo, Giulia Perini, Alfredo Costa, Antonio Pisani, Carlo Morasso, Matteo Cotta Ramusino, Stella Gagliardi

**Affiliations:** 1 Department of Biology and Biotechnology “L. Spallanzani”, University of Pavia, 27100 Pavia, Italy; rosalinda.digerlando01@universitadipavia.it; 2 Molecular Biology and Transcriptomics Unit, IRCCS Mondino Foundation, 27100 Pavia, Italy; francesca.dragoni@mondino.it (F.D.); evelyne.minucchi@mondino.it (E.M.); garofalomaria.bio@gmail.com (M.G.); stella.gagliardi@mondino.it (S.G.); 3 Department of Brain and Behavioral Sciences, University of Pavia, 27100 Pavia, Italy; alfredo.costa@unipv.it (A.C.); or antonio.pisani@mondino.it (A.P.); 4 Unit of Behavioral Neurology and Dementia Research Center, IRCCS Mondino Foundation, 27100 Pavia, Italy; giulia.perini@mondino.it; 5 Unit of Movement Disorders, IRCCS Mondino Foundation, 27100 Pavia, Italy; 6 Istituti Clinici Scientifici Maugeri IRCCS, 27100 Pavia, Italy; carlo.morasso@icsmaugeri.it

**Keywords:** Alzheimer’s disease, Frontotemporal Dementia, microRNAs, extracellular vesicles, fluid biomarkers

## Abstract

Alzheimer’s disease (AD) and Frontotemporal Dementia (FTD) are complex neurodegenerative disorders, often sharing overlapping symptoms. Non-coding RNAs may be involved in pathological processes in these conditions, hence the study of miRNAs isolated from plasma-derived extracellular vesicles (EVs) could provide exploratory insights into the molecular background. The main aim of this work was to identify shared deregulated miRNAs presenting different expression patterns in the two pathologies. A selection of the identified deregulated miRNAs was further studied with the purpose of identifying their mRNA targets and generating hypotheses on their potential pathological involvement. A total of 340 and 291 differentially expressed miRNAs were found in FTD and AD, respectively. Among the commonly deregulated miRNAs with opposite expression patterns between the two conditions, miR-638 emerged as a candidate of interest, showing consistent patterns across our experimental analyses. Nevertheless, these findings are preliminary and intended to be interpreted cautiously, requiring validation in larger cohorts. In addition, the expression of two of its predicted targets in peripheral blood mononuclear cells (PBMCs) appeared to align with miR-638 expression in the same cell type and may reflect potential differences in underlying brain pathological states.

## 1. Introduction

The continuous increase in the elderly population is a major factor contributing to the rising prevalence of age-related neurodegenerative diseases, particularly dementia [1]. Within this spectrum, Alzheimer’s disease (AD) and Frontotemporal Dementia (FTD) account for a substantial burden on the healthcare system [2,3]. Despite their distinct pathological mechanisms, AD and FTD often exhibit overlapping symptoms at onset [4], including cognitive, behavioral and psychological symptoms (BPSD) [5,6,7], which make early diagnosis challenging. This clinical overlap can delay accurate diagnosis, underscoring the need for neuroimaging and fluid-based biomarkers alongside clinical examination [8].

Currently, AD diagnosis is based on the National Institute on Aging-Alzheimer’s Association (NIA-AA) criteria and follows the ATN framework, which integrates clinical, neuropsychological assessment and biomarker measurements reflecting amyloid deposition, tau accumulation and neurodegeneration [9]. In contrast, FTD diagnosis primarily relies on clinical and behavioral features supported by neuropsychological evaluation and structural and functional neuroimaging, according to international consensus criteria [10]. Nevertheless, differential diagnosis between AD and FTD remains challenging, particularly during early disease stages.

In recent years, efforts have been made to identify new biomarkers that could help characterize and, more specifically, distinguish between the two diseases. In this context, different studies have been focusing on the possibility of using extracellular vesicles (EVs) and their cargo as fluid diagnostic biomarkers [11]. In the context of neurodegenerative disorders, EVs released by brain cells in the extracellular space can cross the blood–brain-barrier (BBB) and they can be isolated from peripheral blood, providing a picture of the pathological processes occurring in the brain [12]. Depending on their biogenesis, EVs can be divided into three classes: exosomes (formed intracellularly and released from multivesicular bodies by exocytosis), microvesicles (formed by the outward budding of the cell membrane) and apoptotic bodies (released during cell death) [11]. Alternatively, the 2023 guidelines from the International Society for Extracellular Vesicles (ISEV) classify EVs in two groups: small EVs (SEVs) and large EVs (LEVs) [13].

EVs are natural carriers that can vehicle a variety of molecules, such as proteins, lipids, mRNAs, as well as ncRNAs, among which microRNAs (miRNAs) that when transferred to a recipient cell can mediate target gene silencing [12,14,15]. In particular, miRNAs play a pivotal role in the maintenance of central nervous system (CNS) homeostasis and their dysregulation is linked to an alteration in RNA metabolism and is associated with neurodegeneration [16]. These small non-coding RNAs act by recognizing complementary target sites in the 3′UTR region of target mRNA and by partially binding to these sites can post-transcriptionally modulate gene expression [17]. A previous study explored circulating miRNAs as potential fluid biomarkers by analyzing the cargo of plasma-derived EVs across different neurodegenerative disorders, including AD, FTD, Parkinson’s disease (PD), and Amyotrophic Lateral Sclerosis (ALS) [18]. In this work, both SEVs and LEVs were investigated, revealing disease-related differences in miRNAs content, specifically within the SEV fraction [18]. However, the study primarily provided a descriptive comparison across multiple pathologies and EV subtypes, without focusing on a direct molecular differentiation between clinically overlapping dementias.

Evidence supporting a direct molecular comparison between AD and FTD using a structured multi-step validation framework remains limited. In this context, integrating small RNA sequencing-based discovery with validation across distinct biological compartments may provide a novel approach to understanding whether circulating SEV-derived miRNAs reflect disease-specific mechanisms.

Starting from this, the present study aimed to identify miRNA signatures specifically derived from SEVs that could highlight possible molecular differences between AD and FTD. To this aim, an integrated experimental strategy combining small RNA sequencing, multi-level validation, downstream target analysis, and expanded cohort characterization was implemented.

## 2. Materials and Methods

### 2.1. Cohort Description

Blood samples were collected from AD, FTD and age- and sex-matched controls (CTRL) subjects. Both AD and FTD patients were diagnosed after a clinical and neuropsychological examination at IRCCS Mondino Foundation, following the NIA-AA [8] and the Movement Disorder Society (MDS) [9] diagnostic criteria, respectively. CTRL subjects were recruited at the Transfusion Service and Centre of Transplantation Immunology, San Matteo Foundation, IRCCS (Pavia, Italy). All subjects were included in this study after signing an informed consent form (Protocol no. C.E. 4407/22). The study was conducted in accordance with the ethical principles outlined in the Declaration of Helsinki. Details on the subjects included in the study are reported in Table S5.

### 2.2. SEVs Isolation and Characterization

Venus blood from AD patients (n = 20), FTD patients (n = 12) and CTRL subjects (n = 21) was collected in sodium citrate tubes, and then plasma was retrieved. Differential centrifugation method was applied to separate SEVs from LEVs, following a previously described protocol [18,19]. Briefly, samples were first centrifuged at 20,000× *g* for 1 h at 4 °C to remove LEVs. The resulting supernatant was filtered (0.2 µm) and ultracentrifuged at 100,000× *g* for 1 h at 4 °C. The SEVs-containing pellet was resuspended in PBS and ultracentrifuged again in 100,000× *g* for 1 h at 4 °C, then resuspended in PBS [18,19]. A first characterization step was performed on a representative subset of samples from both EV fractions to evaluate their purity and quality. Nanoparticle Tracking Analysis (NTA) (NanoSight, Malvern, UK) was performed on AD (n = 15), FTD (n = 8) and CTRL (n = 13) samples, while Transmission Electron Microscopy (TEM, Department of Biomedical and Clinical Science, Sacco, Milan, Italy) was performed on AD (n = 2), FTD (n = 2) and CTRL (n = 2) samples to check the obtained LEVs and SEVs fractions. NTA and TEM analyses were conducted on a subset of samples due to limited availability of biological material.

### 2.3. Peripheral Blood Mononuclear Cells (PBMCs) Isolation

PBMCs were isolated from peripheral venous blood of AD patients (n = 20), FTD patients (n = 10) and CTRL subjects (n = 20) using Histopaque^®^-1077 (Sigma-Aldrich, St. Louis, MO, USA) and following manufacturer’s instructions.

### 2.4. RNA Extraction

RNA was extracted from SEV fractions using miRNeasy Mini kit (Qiagen, Hilden, Germany) according to manufacturer’s instructions. Total RNA was then obtained from 5 × 10^6^ PBMC pellets using Trizol reagent (Life Science Technologies, Milan, Italy) according to manufacturer’s instructions. RNA concentration and quality were assessed using NanoDrop™ One/OneC Microvolume UV-Vis Spectrophotometer (Thermo Fisher Scientific, Waltham, MA, USA).

### 2.5. Small RNA Libraries Preparation and Bioinformatic Analysis

Small RNA libraries were prepared starting from RNA extracted from SEVs samples (20 AD, 12 FTD and 21 CTRL) using the Small RNA-Seq Library Prep kit (Lexogen, Wien, Austria). Library quality was assessed with 4200 Tape Station System (Agilent, Santa Clara, CA, USA) and relative High Sensitivity DNA assay (Agilent, USA). Library quantification was performed using the Qubit dsDNA HS Assay Kit (Life Science Technologies, Italy). After multiplexing the individually barcoded libraries, the sequencing steps were carried out on the Illumina NextSeq 500 (Illumina, San Diego, CA, USA).

Starting with raw bcl files, fastq were obtained using bcl2fastq2 Conversion Software v2.20 (Illumina, USA). The trimming, alignment against the reference genome and reads counting were performed with the docker4seq package [20,21]. R package (v. R 2.11) Ebseq was used to perform the differential expression analysis (DEA). Differentially expressed (DE) miRNAs were identified using a threshold of FDR ≤ 0.1 and an absolute log_2_ fold change (|log_2_FC|) > 1, to ensure both statistical relevance and biological significance. The FDR cutoff was set to maintain adequate sensitivity in this exploratory analysis, considering the typically low expression levels of miRNAs and the potential relevance of subtle regulatory changes. Deregulated miRNAs (FDR ≤ 0.1 and |log_2_FC| > 1) identified from miRNA-seq analysis (291 for AD vs. CTRL and 340 for FTD vs. CTRL) were subjected to pathway analysis using miEAA [22] with Over-Representation Analysis (ORA) on the KEGG database, applying FDR (Benjamini-Hochberg) *p*-value adjustment and a significance level of 0.05. The shared DE miRNAs between the two pathologies were visualized using the jvenn viewer [23]. The raw RNA-sequencing data for this manuscript are available in the GEO repository (accession number GSE311885).

### 2.6. Selection and RT-qPCR Validation of DE miRNAs

Among all the DE miRNAs identified, only the 73 miRNAs shared between the two pathological conditions were considered for further analysis. Of these, 38 miRNAs displayed concordant fold-change direction in both conditions, while 35 showed the opposite fold-change direction between AD and FTD. From this subset of 73 shared miRNAs, 7 were selected based on the following criteria:

- Inclusion of miRNAs exhibiting both concordant and discordant expression patterns between AD and FTD;
- A log_2_FC higher than 4 or lower than −4 in at least one of the two conditions;
- Documented in scientific literature.

The 7 identified miRNAs have been validated by RT-qPCR using TaqMan microRNA Assays (ThermoFisher, USA) according to manufacturer’s instructions. These miRNAs were initially validated on SEVs-derived RNA from AD patients (n = 20), FTD patients (n = 28) and CTRL subjects (n = 20). Subsequently, only the 5 miRNAs displaying opposite log_2_FC patterns were validated using PBMCs-derived RNA from AD patients (n = 20), FTD patients (n = 10), and control subjects (n = 20). Candidate miRNAs cycle threshold (Ct) values were normalized against endogenous controls obtained ones. Fold-expression differences between AD or FTD and CTRL groups were determined using the 2^−∆∆Ct^ method. The complete list of validated miRNAs with relative assay IDs is shown in Table 1. The processed dataset can be found at Zenodo repository doi: 10.5281/zenodo.17736953.

### 2.7. Selection and RT-qPCR Validation of Target mRNAs

Starting from the 3 miRNAs (hsa-miR-638, hsa-miR-574-3p and hsa-miR-200a-3p) that showed good results in the previous validation steps and displayed consistent expression patterns across all collected datasets, two target mRNAs were selected for each miRNA. To explore their potential functional roles, miRNet [24] was employed to visualize the interaction network of their predicted target mRNAs. Among all identified targets, the final selection was made by integrating data from TarBase v8 [25] and MirTarBase [26], and according to the following criteria:

- Experimental evidence by a luciferase reporter assay, western blot analysis and RT-qPCR of miRNA-mRNA interaction in human cell lines had to be available;
- Target genes had to show reliable expression levels in PBMCs, according to The Human Protein Atlas database [27];
- Selected mRNAs had to be involved in dementia-related pathways, as determined using the STRING database [28].

Ultimately, 2 target mRNAs were identified for each of the 3 miRNAs under investigation. Starting from PBMCs-derived RNA from AD patients (n = 19), FTD patients (n = 10) and CTRL subjects (n = 20), cDNAs were obtained using the iScript™ cDNA Synthesis Kit (BioRad, Hercules, CA, USA). The iQ™ SYBR^®^ Green Supermix (BioRad, USA) and relative protocol were used to perform RT-qPCR. Primers were designed using Primer3Plus (version 3.3.0) [29]. Candidate targets cycle threshold (Ct) values were normalized against *GAPDH* obtained one. Fold-expression differences between AD or FTD and CTRL groups were determined using the 2^−∆∆Ct^ method. The complete list of validated targets with relative primer sequences is shown in Table S6. The processed dataset can be found at Zenodo repository doi: 10.5281/zenodo.17736953.

It is important to highlight that targets were chosen based on their experimentally validated relationship with the relative miRNA; however, in this study, no functional validation was performed to support these findings. As a consequence, it is fundamental to consider our results only as speculative data.

### 2.8. Statistical Analysis

Figures and statistical analysis were obtained using GraphPad Prism version 9 (USA, RRID: SCR_002798). A paired *t*-test was used to assess whether there was a significant difference in size between the SEVs and LEVs fractions.

Normality of RT-qPCR data for each miRNA and target mRNA across the three groups (AD, FTD, CTRL) was assessed using the Shapiro–Wilk test. Given that the majority of datasets did not meet the assumption of normality, and group sizes were small and unequal in number, all comparisons were performed using non-parametric tests for consistency. Specifically, group differences were assessed using the Kruskal–Wallis test, followed by Dunn’s multiple comparison test. Due to the exploratory nature of this study, limited sample size, and considerable patient-level overlap, Dunn’s multiple comparisons were performed to identify group differences and are interpreted descriptively. Samples showing undetectable or unstable expression (i.e., inconsistent Ct values) were excluded from the analysis. Additionally, outlier detection was performed using GraphPad Prism, and values identified as outliers were removed. Details on excluded samples are provided in Table S4a–e. To evaluate the potential of the validated miR-638 as a pathological discriminative marker in SEVs, ROC curve analyses were performed. The area under the curve (AUC) was calculated to assess the discriminative power of miR-638 in distinguishing disease groups (AD and FTD) from CTRL. Additionally, a specific ROC analysis was conducted for miR-638 to investigate its ability to differentiate between the two pathological conditions (AD vs. FTD). For each ROC curve, the 95% confidence interval (CI) and the *p*-value (null hypothesis AUC = 0.5) were reported. For all analyses, *p* < 0.05 was considered statistically significant. Details on the statistical analysis with relative *p*-values are listed in Table S7a–f.

## 3. Results

In this study, the miRNA expression profile of plasma-derived SEVs was investigated in two neurodegenerative conditions: AD and FTD. Subsequently, attention was directed toward the validation of a subset of the DE miRNAs. Furthermore, starting from the top three validated miRNAs, two experimentally supported targets were selected each and subsequently validated in PBMCs.

### 3.1. SEVs Characterization

**Figure 1:**
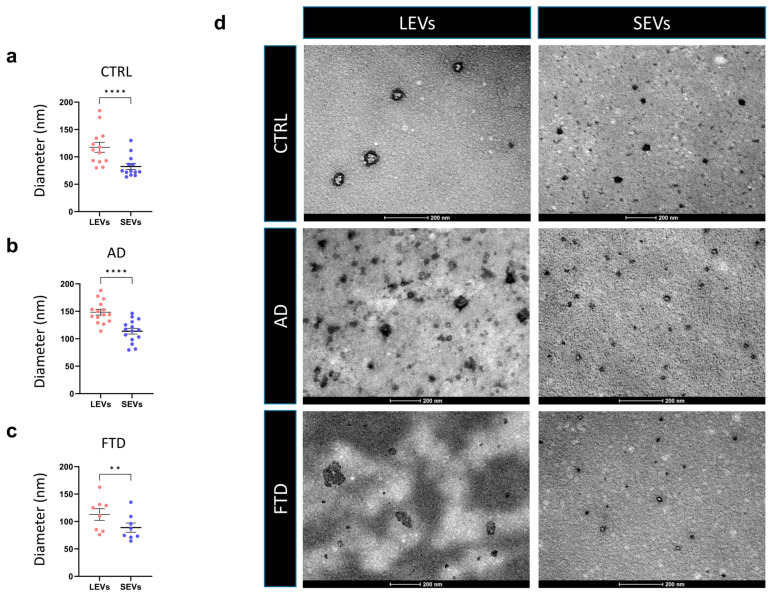
EVs characterization. Size distribution of SEVs and LEVs in (**a**) CTRL (n = 13), (**b**) AD (n = 15), (**c**) FTD (n = 8). X axis: LEVs or SEVs; Y axis: diameter (nm); LEVs and SEVs are differentiated by color. Student’s *t* test: ** *p* < 0.01; **** *p* < 0.0001. Data is expressed as the mean ± SEM. (**d**) Representative images of LEVs and SEVs fractions for each analyzed condition obtained by TEM. Scale bar: 200 nm.

Following differential centrifugation performed according to a previously described protocol [18,19] as detailed in Section 2.2, the size distribution of SEVs and LEVs was assessed in a subset of samples using NTA (Figure 1). As expected, LEVs displayed significantly greater mean diameters compared to SEVs across all analyzed patient groups. Specifically, in CTRL subjects, LEVs showed a mean diameter of 117.54 ± 32.83 nm (mean ± standard deviation, SD), whereas SEVs measured 82.48 ± 19.71 nm (mean ± SD) (Figure 1a). In the AD group, LEVs had a mean diameter of 148.39 ± 20.26 nm (mean ± SD), while SEVs measured 113.81 ± 20.57 nm (mean ± SD) (Figure 1b). Similarly, in FTD patients, the mean diameter of LEVs was 112.71 ± 29.93 nm (mean ± SD), compared to 88.88 ± 23.85 nm (mean ± SD) for SEVs (Figure 1c).

To further support these findings, both SEVs and LEVs fractions for a subset of samples from all three conditions were examined by TEM. Consistently, LEVs displayed particles with visibly larger diameters than SEVs (Figure 1d).

### 3.2. miRNA Expression Profiles in AD and FTD SEVs

**Figure 2:**
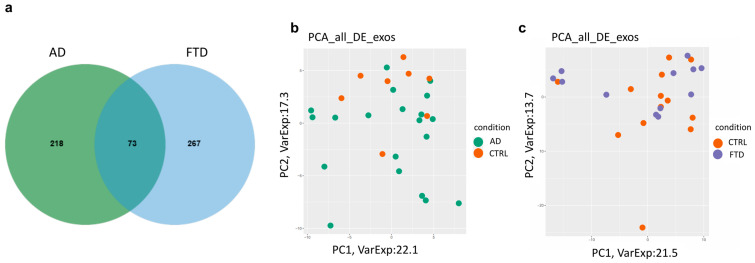
Deregulated miRNAs expression patterns. (**a**) Venn diagram showing common deregulated microRNAs among AD and FTD patients. AD-specific miRNAs are represented in the green circle; FTD-specific miRNAs are represented in the light-blue circle (**b**) PCA analysis of AD patients (n = 20) versus CTRL (n = 8) small RNA sequencing data. AD: green dots; CTRL: orange dots (**c**) PCA analysis of FTD patients (n = 12) versus CTRL (n = 13) small RNA sequencing data. FTD: purple dots; CTRL orange dots.

A previous study reported a more significant enrichment of disease-specific DE miRNAs in SEVs compared to LEVs [18]. Therefore, further analyses were conducted only on SEVs-derived RNA. Initially, DE miRNAs were identified in both the AD and FTD cohorts. A total of 291 and 340 statistically significant DE miRNAs (FDR ≤ 0.1 and |log_2_FC| > 1) were found in AD and FTD patients, respectively (Figure 2a) (Table S1a,b). Specifically, in the AD group, 206 miRNAs were upregulated, and 85 were downregulated, while in the FTD group, 223 miRNAs were upregulated and 117 downregulated (Table 2 and Table S2a,b). Among all DE miRNAs, 73 were found to be shared between the two pathologies. Within this subset, 38 miRNAs displayed concordant log_2_FC direction, while 35 showed opposite log_2_FC direction in AD and FTD (Table 2 and Table S3).

Exploratory data analysis was performed using principal component analysis (PCA) and hierarchical clustering visualized through heatmaps (Figure 2b,c and Figure S1). In the comparison between AD and CTRL groups, CTRL samples clustered predominantly in the upper region of PC2, whereas AD samples displayed a more heterogeneous distribution. The first two principal components, PC1 and PC2, explained 22.1% and 17.3% of the total variance, respectively (Figure 2b). On the contrary, the PCA comparing FTD and CTRL samples did not reveal a clear separation. However, FTD samples tended to cluster toward the upper part of PC2, while CTRL samples showed a more scattered pattern. PC1 and PC2 accounted for 21.5% and 13.7% of the overall variability, respectively (Figure 2c). Heatmap analysis of both comparisons did not reveal a clear expression pattern distinguishing the two groups (Figure S1). PCA and heatmap analyses did not revealed a clear separation between CTRL and the two diagnostic groups, but it is important to acknowledge that in these analyses disease-specific signals from a small panel of miRNAs may be diluted by non-informative variables.

**Figure 3:**
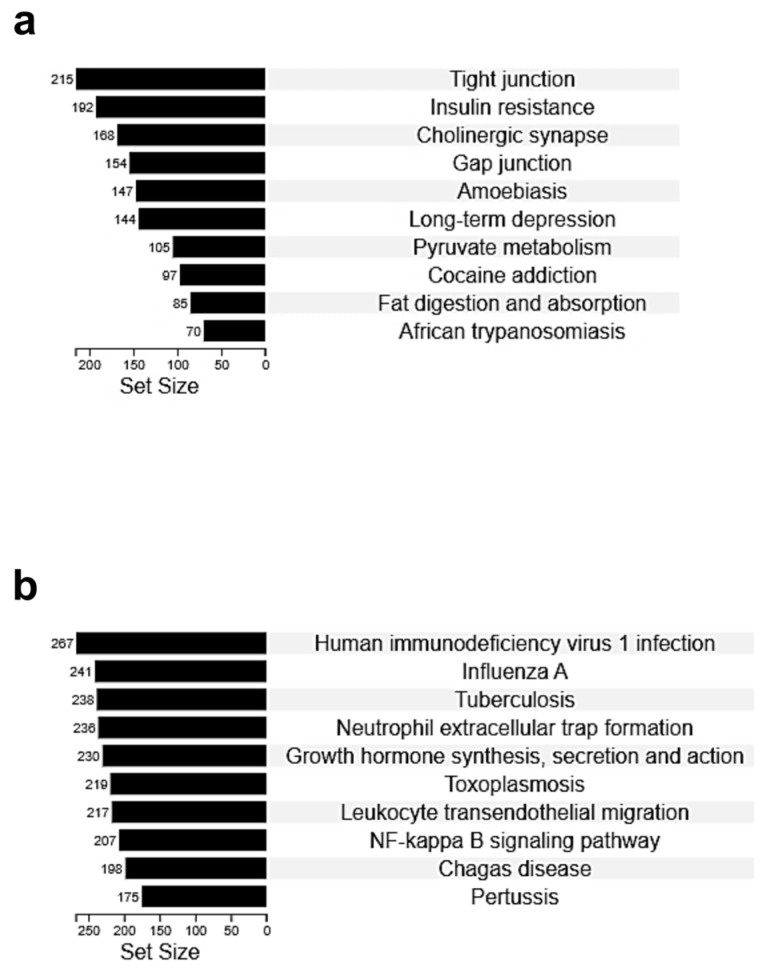
Enrichment analysis of the most significantly enriched KEGG pathways associated with deregulated miRNAs in (**a**) AD and (**b**) FTD. Pathway analysis was performed on DE miRNAs (FDR ≤ 0.1, |log_2_FC| > 1) identified in the miRNA-seq analysis (Table S1) using miEAA [19].

The pathway enrichment analysis presented in Figure 3 was performed on DE miRNAs (FDR ≤ 0.1, |log_2_FC| > 1) identified in the miRNA-seq analysis (Table S1), specifically 291 in AD vs. CTRL and 340 in FTD vs. CTRL. Set size represents the total number of input miRNAs annotated to each pathway. In AD, the most significant pathways were related to neuronal and metabolic processes, including tight junctions, insulin resistance, cholinergic synapses and gap junctions (Figure 3a). In FTD, enriched pathways were predominantly associated with immune response and inflammation, including neutrophil extracellular trap formation and the NF-kβ signaling pathway (Figure 3b). Some non-neurodegenerative pathways emerged likely due to miRNAs’ overlap with immune-related processes.

### 3.3. DE miRNAs Validation in SEVs and PBMCs

**Figure 4:**
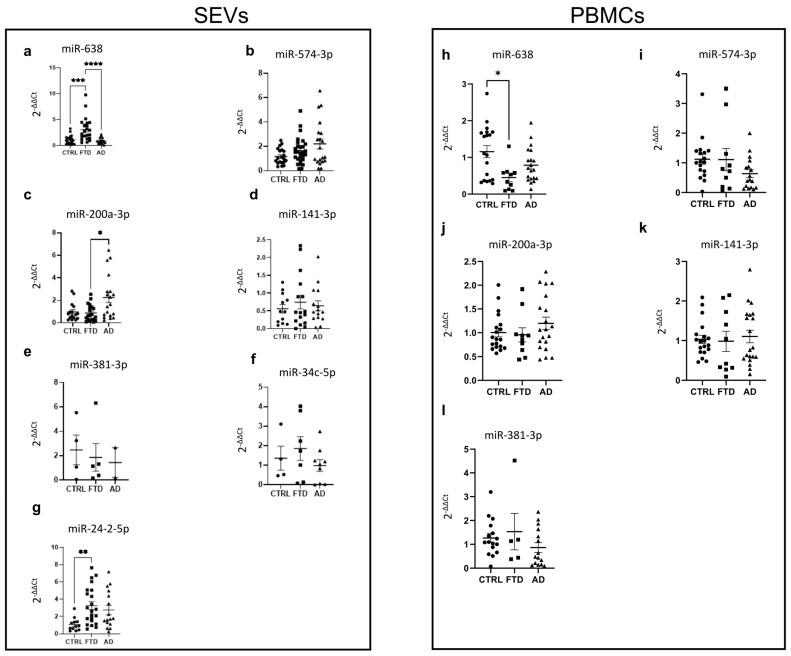
Deregulated miRNAs validation over miR-16 endogenous control. Validation through RT-qPCR of 7 deregulated miRNAs in SEVs from a cohort of AD (n = 20), FTD (n = 28) and CTRL (n = 20). (**a**) miR-638; (**b**) miR-574-3p; (**c**) miR-200a-3p; (**d**) miR-141-3p; (**e**) miR-381-3p; (**f**) miR-34c-5p; (**g**) miR-24-2-5p. Validation through RT-qPCR of 5 deregulated miRNAs in PBMCs from a cohort of AD (n = 20), FTD (n = 10) and CTRL (n = 20). (**h**) miR-638; (**i**) miR-574-3p; (**j**) miR-200a-3p; (**k**) miR-141-3p; (**l**) miR-381-3p. X axis: condition; Y axis: Fold-expression indicated as 2^−∆∆Ct^. Dunn’s multiple comparison: * *p* < 0.05; ** *p* < 0.01; *** *p* < 0.001; **** *p* < 0.0001. Data is expressed as the mean ± SEM. Information on excluded samples is provided in Table S3a–d.

For technical validation, miR-638, miR-574-3p, miR-200a-3p, miR-141-3p, miR-381-3p, miR-24-2-5p and miR-34c-5p were selected among the 73 shared ones based on the criteria defined in the Materials and Methods (Section 2.7). Briefly, these 7 miRNAs were selected as they exhibited both concordant and discordant expression patterns between AD and FTD, presented a log_2_FC higher than 4 or lower than −4 in at least one of the two conditions and were documented in the scientific literature. Among these, 5 miRNAs showed opposite log_2_FC patterns between AD and FTD, while the remaining 2 displayed concordant log_2_FC patterns (Table 1). This initial validation step was performed on RNA extracted from SEVs samples. The expression levels of these miRNAs were normalized using hsa-miR-16 (Figure 4) and U6 snRNA (Figure S2) as endogenous controls, given their widespread use in miRNA studies.

Among the 7 validated miRNAs in EVs, miR-638, miR-574-3p, miR-200a-3p, miR-381-3p and miR-34c-5p exhibited expression patterns consistent with the NGS data under at least one normalization method and for at least one condition. In this section, only the results confirming NGS data are described. Specifically, miR-638 most closely matched the sequencing results for both pathologies when normalized to miR-16, showing downregulation in the AD group and significant upregulation in FTD with respect to CTRL (Figure 4a). The upregulation in FTD was also confirmed as statistically significant upon normalization to U6 snRNA (Figure S2a). Additionally, miR-574-3p showed upregulation in AD, while miR-381-3p and miR-34c-5p exhibited downregulation and upregulation in FTD, respectively, with coherent expression patterns confirmed using both normalization methods (Figure 4b,e,f and Figure S2b,e,f). On the other hand, miR-200a-3p showed upregulation in AD with both normalization methods (Figure 4c and Figure S2c), while miR-141-3p displayed non-significant upregulation in AD (Figure 4d). The miRNA that did not corroborate the transcriptomic results under either normalization method was miR-24-2-5p (Figure 4g and Figure S2g).

**Figure 5:**
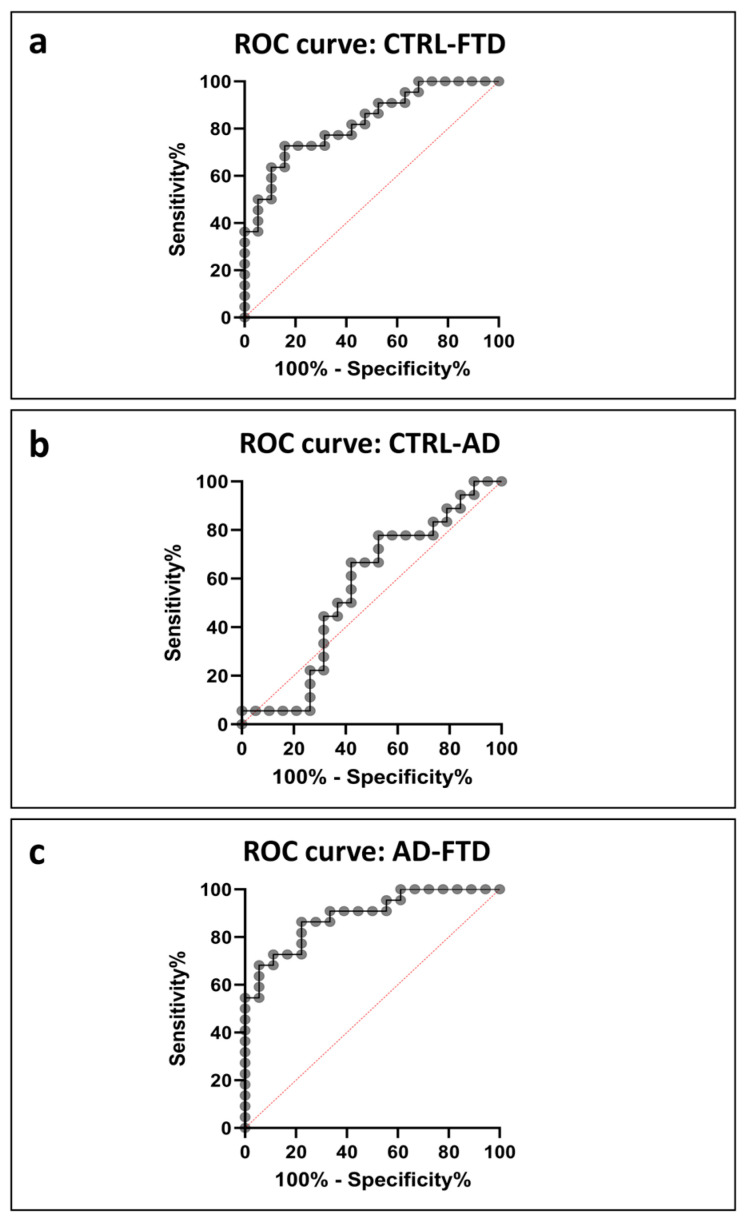
ROC curve analysis of miR-638 qPCR validations in SEVs. (**a**) FTD vs. CTRL (AUC = 0.8254; 95% CI: 0.7009–0.9498; *p* = 0.0004); (**b**) AD vs. CTRL (AUC = 0.5556; 95% CI: 0.3635–0.7476; *p* = 0.5637); (**c**) AD vs. FTD (AUC = 0.8889; 95% CI: 0.7909–0.9869; *p* < 0.0001). X axis: specificity %; Y axis: sensitivity %. The red line represents a baseline of random guessing.

To further investigate the potential of miR-638 as a fluid biomarker, ROC curve analyses were performed on RT-qPCR data normalized to miR-16. In the FTD group, miR-638 demonstrated a robust discriminative capacity compared to CTRL, with an AUC of 0.8254 (95% CI: 0.7009–0.9498, *p* = 0.0004; Figure 5a). Conversely, miR-638 showed poor diagnostic performance in distinguishing AD from CTRL, yielding an AUC of 0.5556 (95% CI: 0.3635–0.7476, *p* = 0.5637; Figure 5b). Notably, when tested for its differential diagnostic potential, miR-638 exhibited its highest performance in discriminating FTD from AD patients, with an AUC of 0.8889 (95% CI: 0.7909–0.9869, *p* < 0.0001; Figure 5c). These results identify miR-638 as a highly specific candidate for FTD, reinforcing its potential utility in the differential diagnosis of dementia-related pathologies.

The second validation step was performed on RNA isolated from PBMCs. Only the 5 miRNAs displaying opposite log_2_FC patterns (miR-638, miR-574-3p, miR-200a-3p, miR-141-3p, miR-381-3p) were validated to assess their expression levels in a blood-derived cell population (Table 1). Data normalization was carried out as previously described, using hsa-miR-16 (Figure 4) and U6 snRNA (Figure S2) as endogenous controls, given their widespread use in miRNA studies.

In this section, only the results confirming NGS data are described.

It was found that miR-638 was downregulated in AD only when normalized to miR-16 (Figure 4h). Similarly, miR-574-3p was upregulated in AD with U6 normalization (Figure S2i). Both miR-200a-3p and miR-141-3p showed similar patterns, being upregulated in AD with both normalization methods and downregulated in FTD only when normalized to miR-16 (Figure 4j,k and Figure S2j,k). Lastly, miR-381-3p matched the NGS data only in AD samples normalized to U6 snRNA (Figure S2l).

### 3.4. Target Selection and Validation in PBMCs

**Figure 6:**
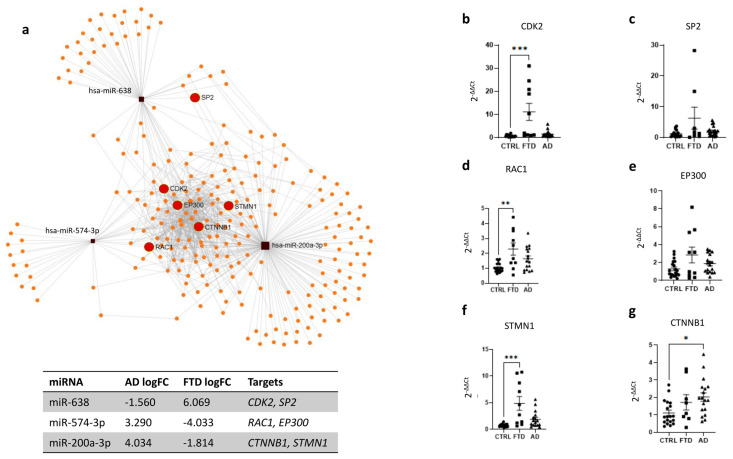
Targets identification and analysis. (**a**) miRNAs-mRNAs network miRNet [20] visualization. Validation through RT-qPCR of the selected targets in PBMCs from a cohort of AD (n = 19), FTD (n = 10) and CTRL (n = 20). (**b**) *CDK2*; (**c**) *SP2*; (**d**) *RAC1*; (**e**) *EP300*; (**f**) *STMN1*; (**g**) *CTNNB1*. X axis: condition; Y axis: Fold-expression indicated as 2^−∆∆Ct^. Dunn’s multiple comparison: * *p* < 0.05; ** *p* < 0.01; *** *p* < 0.001. Data is expressed as the mean ± SEM. Information on excluded samples is provided in Table S4e.

Taking everything together, the miRNAs displaying the best results in terms of consistency between NGS data and the two validation steps, as well as the total number of analyzed samples, were miR-638, miR-574-3p and miR-200a-3p. To better understand their function and role under pathological conditions, two target genes were selected for each miRNA, and their expression was assessed in PBMCs. Among all predicted targets, the final selection was carried out following the criteria described in the Materials and Methods (Section 2.7), namely the availability of experimental evidence supporting miRNA-mRNA interaction in human cell lines, reliable expression of target genes in PBMCs, and involvement of the selected genes in dementia-related pathways. Figure 6a shows the miRNA-target network together with a small table reporting the log_2_FC for the three miRNAs and the two targets selected for each one of them.

For miR-638, the two identified targets were *CDK2* and *SP2*. Both targets were found to be upregulated mostly in FTD (Figure 6b,c). Although not coherent with NGS results, these findings were consistent with the downregulation of miR-638 observed in PBMCs and suggest a potential regulatory interaction.

As for miR-574-3p, *RAC1* and *EP300* were chosen as targets. Both genes were upregulated under pathological conditions (Figure 6d,e) matching the miRNAs expression in PBMCs and supporting a possible inverse relationship.

Lastly, *CTNNB1* and *STMN1* were selected as miR-200a-3p targets. Both appeared upregulated in AD and FTD (Figure 6f,g). These last results appeared to be coherent with miR-200a-3p expression levels in FTD, but not in AD.

## 4. Discussion

Finding novel biomarkers that may help define the molecular pathological characteristics of neurodegenerative diseases is crucial. This is particularly true for those cases where clinical symptoms can be similar, like AD and FTD. EVs, and more specifically the miRNAs they carry, may be useful in this context. Indeed, being easily retrievable from peripheral blood, they represent promising candidates as novel fluid biomarkers. In this study, SEVs-derived miRNAs were analyzed and validated to explore their potential role and the involvement of their targets in these two pathologies. Moreover, EVs-derived miRNAs were compared with PBMCs-derived miRNAs to get information about the vesicular component and the cellular component, respectively, to check if there was any overlapping miRNA signature and obtain complementary aspects of the disease molecular biology.

Following the initial characterization, SEV-derived miRNAs were sequenced. Although PCA analysis did not reveal clear disease-specific clustering, the deregulated miRNAs identified in this study were associated with neurodegeneration-relevant pathways, supporting the potential biological relevance of our findings. Specifically, pathway analysis revealed that miRNAs deregulated in AD were enriched in neuronal and metabolic processes, such as tight junctions, insulin resistance, cholinergic synapses and gap junctions [25,26]. In contrast, miRNAs deregulated in FTD were more strongly associated with inflammatory processes, highlighting pathways such as neutrophil extracellular trap formation and the NF-kβ signaling pathway [30]. Notably, FTD analysis also highlighted non-neurodegenerative pathways, reflecting miRNA enrichment biases toward high-study fields and miRNAs’ pleiotropy in immune responses, consistent with FTD neuroinflammation [30]. Moreover, since the EVs were isolated from plasma, systemic inflammatory signals are likely to contribute to the observed enrichment patterns. These AD/FTD pathway differences suggest a distinct molecular background between the two pathologies and indicate that these different pathways could contribute to defining disease-specific molecular signatures.

By integrating the NGS data with the validation steps, the most significant outcome of this study is the identification of three key miRNAs, namely miR-638, miR-574-3p, miR200a-3p, along with their respective target genes, highlighting their potential role in dementia-related pathologies.

Concerning miR-638, no studies have directly linked it to AD or FTD, at least to our knowledge. Nevertheless, it has been reported to be transferred by EVs and dysregulated in ALS patients [31]. The same group also observed an inverse expression pattern between miR-638 and its host gene, *DNM2*, a gene known for its role in membrane trafficking [31].

In the present study, miR-638 was found to be downregulated in AD in NGS data, and this direction was confirmed across validation steps, hinting at a potential involvement of this miRNA in the disease. However, ROC analysis revealed that this miRNA SEVs concentration lacks sufficient power to reliably discriminate AD from CTRL (AUC = 0.55), suggesting that its involvement in AD may be less specific or more influenced by individual variability. In contrast, miR-638 appeared upregulated in FTD, in both transcriptomic data and SEVs validation, showing a robust discriminative capacity (AUC = 0.82, *p* < 0.001). Most notably, miR-638 exhibited a good potential in the differential diagnosis between the two pathologies, providing a separation of FTD from AD patients with an AUC of 0.89. This finding is particularly relevant as it suggests that miR-638 could serve as an SEV-derived candidate to distinguish AD from FTD; however, this study is only exploratory and further validations are required to confirm this diagnostic hypothesis. Concerning miR-638 expression in FTD PMBCs, downregulation was also observed. The absence of consistent expression patterns between SEVs and PBMCs in FTD may reflect the distinct biological information conveyed by these compartments. SEVs, potentially enriched in central nervous system (CNS)-derived vesicles, may partially mirror molecular processes occurring within the CNS [12]. In contrast, PBMCs represent peripheral immune cells and are therefore more likely to reflect systemic or inflammation-related transcriptional changes. Hence, the limited concordance between SEVs and PBMCs miRNA expression profiles may thus suggest that circulating miRNAs and cellular miRNAs provide complementary information, with SEVs-derived miRNAs potentially more closely linked to neurodegenerative mechanisms, and PBMCs-derived miRNAs reflecting systemic pathological responses. This hints at a possible SEV-specific regulation of miR-638.

Research was carried out on miR-638 by integrating data from the Tarbase v.8 [32] and MirTarBase [33] databases with the aim of identifying its targets. Only those interactions studied in vitro in human cell lines and experimentally validated were selected. Two genes were found to match these criteria and appeared to be linked to miR-638: *SP2* and *CDK2*. *SP2* is a transcription factor described as a potential regulator of genes forming the common neurodegeneration module (CNM) [34]. These genes are involved in key pathological pathways, such as glial activation, neuroinflammation, mitochondrial dysfunction, and protein aggregation [35]. *SP2* marked upregulation in FTD may underline its early synaptic alteration, and it may be a compensatory response to neuronal disfunction [36]. In AD, its lower expression may result from the advanced Aβ accumulation and more pronounced atrophy levels [36]. On the other hand, *CDK2* is a kinase that regulates cell cycle progression and germ cell development [37]. Under pathological conditions, different cyclin-dependent kinases have been implicated as potential contributors to neurodegenerative processes, as their increased expression has been observed to precede both Tau protein hyperphosphorylation and Aβ accumulation in cellular and mouse models of AD [38]. Also in this case, *CDK2* appears to be upregulated in FTD, which may represent a pathological event preceding Tau accumulation. By contrast, at the time of diagnosis, AD usually presents with both Aβ and Tau pathology [39], so *CDK2* expression may me milder.

The fact that in this study both targets were found to be upregulated in AD and even more markedly in FTD is consistent with the observed downregulation of miR-638 in PBMCs. The discrepancy between the expression of the investigated targets in our study and some reports in the literature could be explained by the fact that we were assessing gene expression in peripheral cells, such as PBMCs, rather than in CNS tissues.

Furthermore, the two target expression patterns may also be related to the distinct patterns of neuronal atrophy in the two pathologies. Indeed, at the time of diagnosis, AD is usually associated with a more widespread severe neuronal loss, specifically affecting the hippocampus, parietal and temporal lobes, while FTD shows greater atrophy in the frontal and temporal lobes [36]. The more generalized synaptic degeneration in AD [40] might contribute to a lower overall transcriptional output, including fewer upregulated genes [41]. In peripheral tissues, similar trends have been reported, with more differentially expressed mRNAs and miRNAs in FTD than in AD [18], reflecting the CNS condition. This observation is generally consistent with our miRNA-seq data, showing 340 DE miRNAs in FTD and 291 in AD. It also reinforces the potential of miR-638 as a specific biomarker for FTD, as it presents a distinct expression profile compared to AD (upregulation in FTD SEVs vs. downregulation in AD SEVs). Consequently, this pattern could also explain the generalized increase in expression of five out of six validated targets.

As for miR-574-3p, the NGS data were validated in AD SEVs, while in FTD SEVs, this miRNA displayed opposite results between NGS and RT-qPCR data. This discrepancy may reflect the limited statistical power associated with smaller FTD sample sizes, which may increase sensitivity to inter-individual variability between discovery and validation datasets. As for PBMCs, miR-574-3p displayed a downregulation in AD and no differential expression in FTD. Discrepancies between NGS and RT-qPCR results may also be influenced by the adopted normalization strategy. In this context, the identification of stable endogenous controls remains challenging, particularly when analyzing distinct biological compartments. U6, although commonly used in cellular RNA analyses, may exhibit variable abundance in EVs due to its nuclear origin [42]. Conversely, miR-16 is frequently used in both biofluid and cellular studies [43]. Accordingly, different endogenous controls were employed based on the biological nature of SEVs and PBMCs. Moreover, given the low RNA input of SEV fractions, even minor fluctuations in reference expression may significantly affect relative quantification, potentially influencing fold-change estimation and biological interpretation.

To date, the function of miR-574-3p, as well as its potential involvement in AD, FTD or other neurodegenerative diseases, has not been fully elucidated. Among its predicted targets, RAC1 and EP300 were selected and validated. Both were found to be upregulated in AD and FTD. Notably, their upregulation was coherent only with the expression pattern of miR-574-3p in PBMCs, and not in SEVs. This difference may once again be explained by the difference in the two sample types. RAC1 is a member of the Rho GTPase family, and it has been associated with AD. Indeed, it contributes to the loss of the protein domain network (PDN), a mechanism responsible for neurodegeneration [44]. A recent study reported that RAC1 expression following two distinct patterns depending on AD stage. Specifically, it is overexpressed in the early stages, where it is directly associated with Aβ and Tau, but it becomes less expressed or downregulated in later stages [45]. In our data, the marked increase observed in FTD may reflect the fact that brain atrophy in FTD is generally less pronounced than in AD [36]. On the other hand, EP300 is a histone acetyltransferase that mediates the acetylation of AD-related gene promoters, such as PS1 or BACE1, leading to their transcription activation [46]. Although EP300 role in AD and FTD remains unclear, its dysregulation in dementia may reflect an alteration in epigenetic regulation in both pathologies.

Lastly, miR200a-3p expression was consistently deregulated across SEVs and PBMCs in both pathologies, most prominently in AD. This miRNA has previously been reported in the literature as associated with AD, and among its predicted targets is *BACE1*, a key enzyme involved in the amyloidogenic proteolysis of *APP* [47]. In this study, *CTNNB1* and *STMN1* were selected and validated. Both were found to be upregulated in AD and FTD. These results align with the miR-200a-3p expression pattern in both SEVs and PBMCs.

Specifically, *STMN1* gene encodes for a ubiquitous protein that, under pathological conditions, contributes to microtubule destabilization and subsequent neurodegeneration [48]. In our data, this gene resulted in being upregulated in both diseases, presenting a marked overexpression in FTD. This pronounced upregulation in FTD may reflect a pathological response to the early neurodegenerative changes occurring in the disease [36]. By contrast, in AD, where neurodegeneration is more severe and widespread, the upregulation is less pronounced, possibly because the affected brain tissue is already undergoing extensive degeneration [36]. Conversely, *CTNNB1*, which encodes for β-catenin, takes part in the Wnt/β-catenin signaling pathway, whose dysregulation has been linked to neurodegeneration [49]. *CTNNB1* appeared upregulated in both diseases, specifically AD. Oxidative stress and neuroinflammation, two key hallmarks of neurodegeneration, are known to promote β-catenin phosphorylation and degradation [50], which may lead to an increase in its expression proportional to its degradation and the intensity of these stressors. In this framework, β-catenin upregulation may represent a compensatory protective mechanism aimed at promoting neuronal survival, maintaining synaptic plasticity, and preserving neuronal integrity [51]. Accordingly, the higher expression observed in AD compared to FTD could be explained by the more severe and widespread neurodegeneration characterizing AD, whereas the milder phenotype of FTD is associated with a less pronounced response.

Taken together, our data suggests that among the 35 miRNAs shared between AD and FTD but showing opposite expression patterns, miR-638 may play a role in dementia, and may be considered as a potential interesting SEVs-specific differential marker in discriminating between AD and FTD. Nevertheless, further validation in a larger cohort is needed to confirm its possible diagnostic role, at least in FTD. Regarding miR-574-3p and miR-200a-3p, validations suggested their dysregulation in AD. It is important to highlight that this study does not aim to replace the traditional biomarkers such as Aβ40, Aβ42, pTau, NfL or imaging techniques. These established markers provide a strong clinical validation and diagnostic accuracy; however, their invasiveness and cost might represent a limitation. In this context, the need for minimally invasive biomarkers has emerged and SEV-associated miRNAs represent a good candidate. In fact, they might offer complementary information giving their accessibility and potential ability to reflect disease-related molecular alterations. On the other hand, miRNA expression may be influenced by pre-analytical variables, comorbidities, sample processing methods and limited reproducibility across independent cohorts, which can contribute to variability across different studies. Future works should evaluate whether integrating miRNA signatures with established biomarkers could improve diagnostic stratification and disease monitoring.

In conclusion, this exploratory study identified two miRNAs not previously associated with dementia (miR-638 and miR-574-3p) and validated one already described in the literature (miR-200a-3p). Although these findings highlight the potential relevance of SEVs-derived miRNAs as candidate fluid biomarkers, the present study is exploratory. Indeed, the identified signatures require validation in larger, independent cohorts to confirm their robustness and possible clinical applicability. Therefore, these results are intended as hypotheses rather than evidence of diagnostic utility.

## 5. Limitations

Despite the potential value of the three miRNAs (miR-638, miR-574-3p, miR-200a-3p), some challenges remain. First, differences in the cohort composition between the NGS and RT-qPCR analyses, including the number of individuals, could explain some inconsistent results. In particular, the modest sample size, especially within the FTD cohorts (considering that FTD is a rare disease and has a heterogeneous clinical presentation), may have limited the statistical power to detect appreciable effect sizes, increasing the likelihood of variability between discovery and validation steps. This reduced power may have contributed to the inconsistencies observed between NGS and RT-qPCR results, particularly for miRNAs showing borderline expression changes. However, this study is intended to be only exploratory and a larger cohort for each diagnostic group, especially FTD, needs to be recruited for future validations in order to get a more robust results that can be generalized. Moreover, the choice of endogenous controls for RT-qPCR normalization represents an additional potential source of variability. Indeed, the stability of U6 and miR-16 may differ between SEVs and PBMCs due to their distinct biogenesis, abundance, and susceptibility to pre-analytical variation. Literature shows a widespread use of these endogenous controls; however, in miRNA studies, normalization is a well-known challenge given the unstable nature of small RNAs. This may affect the reliability of differential expression results; as a consequence, in this work, we focused on miRNAs that had a consistent trend between miR-16 and U6 normalization in at least one tissue. Another important limitation is the low amount of RNA present in SEVs, which may have resulted in the underrepresentation of certain miRNAs in NGS data due to the low number of sequencing reads. Also, differences in tissue type, SEVs versus PBMCs, may greatly contribute to variability in expression patterns observed between these sample types. Finally, while Dunn’s post-hoc testing identified statistically significant pairwise differences, considerable patient-level overlap confirms these results should be considered exploratory.

## Figures and Tables

**Table 1:** List of validated miRNAs.

miRNA Name	Assay ID	AD log_2_FC	FTD log_2_FC	log_2_FC Direction	miRNA Type
hsa-miR-638	001582	−1.56	6.07	miRNAs with opposite direction	candidate miRNA
hsa-miR-574-3p	002349	3.29	−4.03		candidate miRNA
hsa-miR-200a-3p	000502	4.03	−1.81		candidate miRNA
hsa-miR-141-3p	000463	5.41	−1.46		candidate miRNA
has-miR-381-3p	000571	5.59	−3.57		candidate miRNA
hsa-miR-24-2-5p	002441	−4.67	−1.57	miRNAs with concordant direction	candidate miRNA
hsa-miR-34c-5p	000428	4.36	3.71		candidate miRNA
U6 snRNA	001973	-	-	-	endogenous control
hsa-miR-16	000391	-	-	-	endogenous control

log_2_FC = log_2_ fold change.

**Table 2:** Number of statistically significant DE miRNAs in SEVs from AD and FTD patients.

DE miRNAs	AD	FTD
Total number of DE miRNAs	291	340
Number of upregulated miRNAs	206	223
Number of downregulated miRNAs	85	117
Number of shared miRNAs	73	
miRNAs with concordant log_2_FC pattern	38	
miRNAs with the opposite log_2_FC pattern	35	

miRNAs were considered DE when |log_2_$\frac{AD\;or\;FTD}{CTRL}$| > 1 and FDR < 0.1. miRNA = microRNA; AD = Alzheimer Disease; FTD = Fronto-Temporal Dementia; FDR = False Discovery Rate.

## Data Availability

The original data presented in the study are openly available in Zenodo at doi: 10.5281/zenodo.17736953. The RNA-sequencing data for this manuscript are available in the GEO repository (accession number GSE311885).

## Supplementary Materials

The following supporting information can be downloaded at: https://www.mdpi.com/article/10.3390/cimb48060633/s1.

## Abbreviations

AD	Alzheimer’s disease
FTD	Frontotemporal Dementia
BPSD	Behavioral and psychological symptoms
EVs	Extracellular vesicles
BBB	Blood–brain barrier
SEVs	Small extracellular vesicles
LEVs	Large extracellular vesicles
ISEV	International Society for Extracellular Vesicles
miRNAs	MicroRNAs
CNS	Central nervous system
PD	Parkinson’s disease
ALS	Amyotrophic Lateral Sclerosis
CTRL	Control subjects
NIA-AA	National Institute on Aging Alzheimer’s Association
MDS	Movement Disorder Society
PBMCs	Peripheral blood mononuclear cells
NTA	Nanoparticle tracking analysis
TEM	Transmission electron microscopy
DEA	Differential expression analysis
DE	Differentially expressed
FDR	False discovery rate
PCA	Principal component analysis
Ct	Cycle threshold
