# Supplementary material for: Plasma-Derived miRNAs as Fluid Biomarkers to Differentiate Alzheimer’s and Frontotemporal Dementia
Source: Curr Issues Mol Biol. 2026 Jun 17;48(6):633. doi: 10.3390/cimb48060633 (PMC13298512; doi:10.3390/cimb48060633)
Supplement: Supplementary file 1 [file cimb-48-00633-s001.zip › cimb-4295870-Figures S1 and S2.pdf]

Plasma-derived miRNAs as fluid biomarkers to differentiate Alzheimer’s and Frontotemporal Dementia

Rosalinda Di Gerlando [1,2], Evelyne Minucchi [2,3], Francesca Dragoni [2], Maria Garofalo [2], Giulia Perini [4], Alfredo Costa [3,4], Antonio Pisani [3,5], Carlo Morasso [6], Matteo Cotta Ramusino [4*] and Stella Gagliardi [2]

[1]Department of Biology and Biotechnology “L. Spallanzani”, University of Pavia, 27100 Pavia, Italy;

[2]Molecular Biology and Transcriptomics Unit, IRCCS Mondino Foundation, 27100 Pavia, Italy;

[3]Department of Brain and Behavioral Sciences, University of Pavia, 27100 Pavia, Italy;

[4]Unit of Behavioral Neurology and Dementia Research Center, IRCCS Mondino Foundation, 27100 Pavia, Italy;

[5]Unit of Movement Disorders, IRCCS Mondino Foundation, Pavia, 27100 Pavia, Italy;

[6]Istituti Clinici Scientifici Maugeri IRCCS, Pavia, 27100, Italy;

* Correspondence: corresponding author email: matteo.cottaramusino@mondino.it

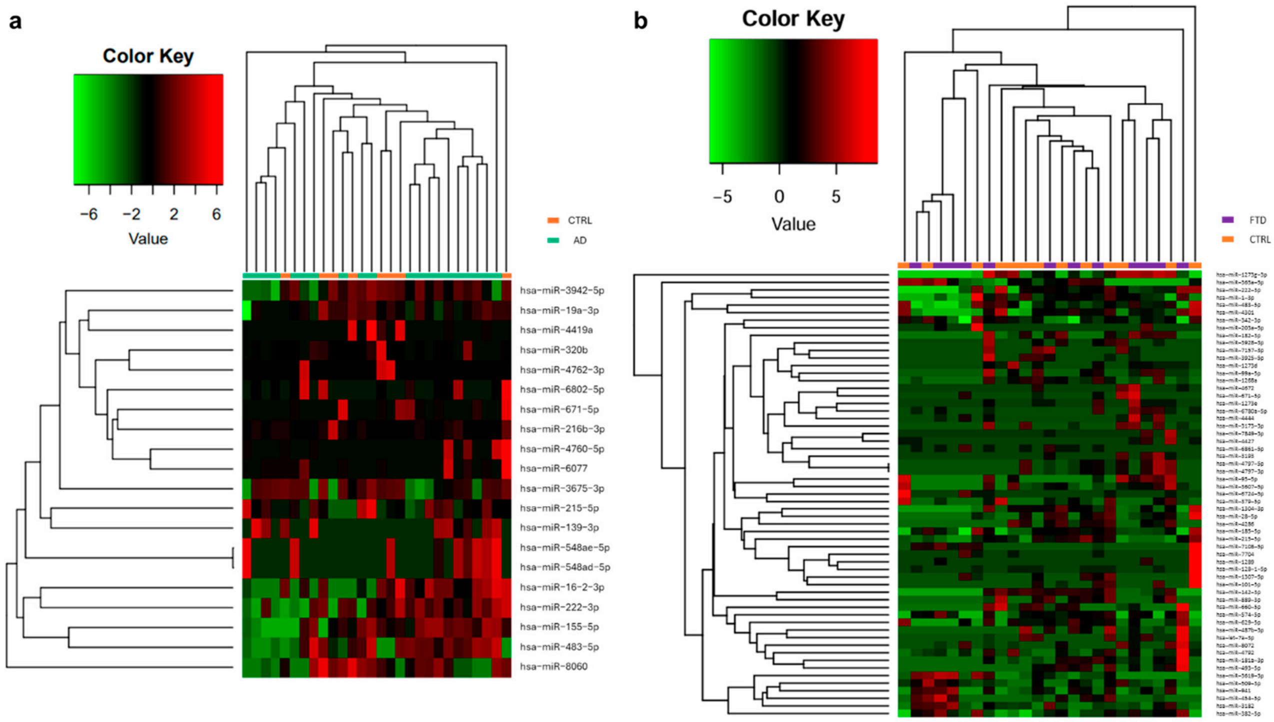

Figure S1. Hierarchical clustering heatmap of deregulated miRNAs across AD, FTD, and control samples. (**a**) AD (n = 20) versus CTRL (n = 8) heatmap. (**b**) FTD (n = 12) versus CTRL (n = 13) heatmap. Rows represent miRNAs, columns represent individual samples; AD: green; CTRL: orange; FTD: purple. Expression values are log_2-transformed and normalized.

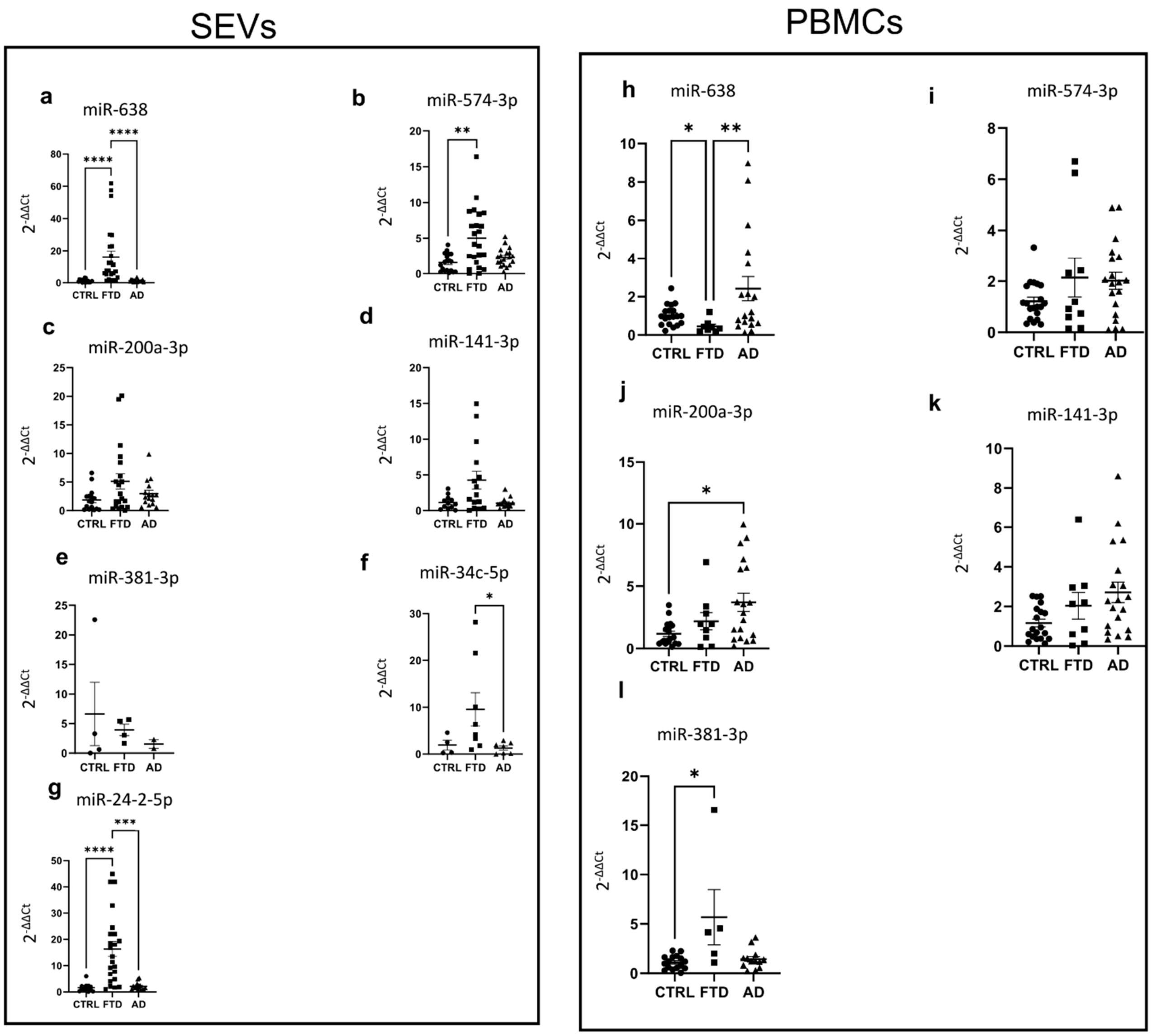

Figure S2. Deregulated miRNAs validation over U6 snRNA endogenous control. Validation through RT-qPCR of 7 deregulated miRNAs in SEVs from a cohort of AD (n=20), FTD (n=28) and CTRL (n=20). (**a**) miR 638; (**b**) miR-574-3p; (**c**) miR-200a-3p; (**d**) miR-141-3p; (**e**) miR-381-3p; (**f**) miR-34c-5p; (**g**) miR-24-2-5p. Validation through RT-qPCR of 5 deregulated miRNAs in PBMCs from a cohort of AD (n=19), FTD (n=10) and CTRL (n=20). (**h**) miR 638; (**i**) miR-574-3p; (**j**) miR-200a-3p; (**k**) miR-141-3p; (**l**) miR-381-3p. X axis: condition; Y axis: Fold-expression indicated as $2^{-\Delta\Delta Ct}$. One-way ANOVA Kruskal-Wallis test: * $p<0.05$; ** $p<0.01$; *** $p<0.001$; **** $p<0.0001$. Data is expressed as the mean ± SEM. Information on excluded samples is provided in Table S4a-d.
